# Anti-inflammatory Activity of the Protein Z-Dependent Protease Inhibitor

**DOI:** 10.1055/s-0041-1730037

**Published:** 2021-06-25

**Authors:** Mahita Razanakolona, Frédéric Adam, Elsa Bianchini, François Saller, Allan de Carvalho, Jean-Luc Diehl, Cécile V. Denis, Ferhat Meziani, Delphine Borgel, Julie Helms, Marc Vasse

**Affiliations:** 1 HITh, INSERM, UMR_S1176, Université Paris-Saclay, Le Kremlin-Bicêtre cedex, France; 2 Département de réanimation médicale, Hôpital Européen Georges Pompidou, Paris, France; 3 Faculté de Médecine, Service de Médecine Intensive-Réanimation, Université de Strasbourg (UNISTRA), Hôpitaux Universitaires de Strasbourg, Nouvel Hôpital Civil, Strasbourg, France; 4 INSERM (French National Institute of Health and Medical Research), Regenerative Nanomedicine (RNM), FMTS, Strasbourg, France; 5 APHP, Laboratoire d'Hématologie, Hôpital Universitaire Necker-Enfants Malades, Paris, France; 6 ImmunoRhumatologie Moléculaire, LabEx TRANSPLANTEX, Centre de Recherche d'Immunologie et d'Hématologie, Faculté de Médecine, Fédération Hospitalo-Universitaire (FHU) OMICARE, Fédération de Médecine Translationnelle de Strasbourg (FMTS), Université de Strasbourg, Strasbourg, France; 7 Service de Biologie Clinique, Hôpital Foch, Suresnes, France

**Keywords:** protein Z-dependent plasma protease inhibitor, proinflammatory cytokines, CCL5, lipopolysaccharide, monocytes

## Abstract

The protein Z (PZ)-dependent plasma protease inhibitor (ZPI) is a glycoprotein that inhibits factor XIa and, in the presence of PZ, FXa. Recently, ZPI has been shown to be an acute-phase protein (APP). As usually APPs downregulate the harmful effects of inflammation, we tested whether ZPI could modulate the increase of cytokines observed in inflammatory states. We observed that recombinant human ZPI (rhZPI) significantly decreases the levels of interleukin (IL)-1, IL-6, and tumor necrosis factor- α (TNF-α) induced by lipopolysaccharide (LPS) in a whole blood model. This inhibitory effect was unaffected by the presence of PZ or heparin. A ZPI mutant within the reactive loop center ZPI (Y387A), lacking anticoagulant activity, still had an anti-inflammatory activity. Surprisingly, rhZPI did not inhibit the synthesis of IL-6 or TNF-α when purified monocytes were stimulated by LPS, whereas the inhibitory effect was evidenced when lymphocytes were added to monocytes. The requirement of lymphocytes could be due to the synthesis of CCL5 (RANTES), a chemokine mainly produced by activated lymphocytes which is induced by rhZPI, and which can reduce the production of proinflammatory cytokines in whole blood. Lastly, we observed that the intraperitoneal injection of rhZPI significantly decreased LPS-induced IL-6 and TNF-α production in mouse plasma.

## Introduction

The protein Z-dependent plasma protease inhibitor (ZPI) is a member of the serpin superfamily identified in human plasma that produces rapid inhibition of factors Xa (FXa) and XIa (FXIa). 1 The inhibition of FXa requires the presence of protein Z (PZ), a vitamin K-dependent factor, which increases more than 1,000-fold the inhibition rate of FXa. In contrast, the rate and extent of FXIa inhibition by ZPI are reduced by the binding of PZ to ZPI, whereas it is enhanced in the presence of unfractionated heparin (UFH). During its inhibitory interaction with FXa and FXIa, ZPI is proteolytically cleaved with the release of a 4.2-kDa peptide. 2 PZ and ZPI circulate as a complex in both human and mouse plasma, but in humans, there is an excess in free ZPI, whereas in mice there is an excess of free PZ. 3 4 ZPI-deficient mice exhibit a higher frequency of thrombosis following arterial injury and an increased mortality from pulmonary thromboembolism following collagen/epinephrine infusion than wild-type (WT) mice. 5 However, the consequences of a ZPI deficiency in humans remain uncertain. 6 7

Recently, it was shown that subcutaneous injection of turpentine in mice induced an approximately threefold increase in both ZPI mRNA and plasma levels, indicating that ZPI is an acute-phase reactant. 4 In contrast, PZ mRNAs were not affected, and the twofold increase of plasma PZ levels, which appeared 2 days after the increase of plasma ZPI levels, was attributed to a prolongation of the PZ half-life when it circulates in complex with ZPI.

**Fig. 1:**
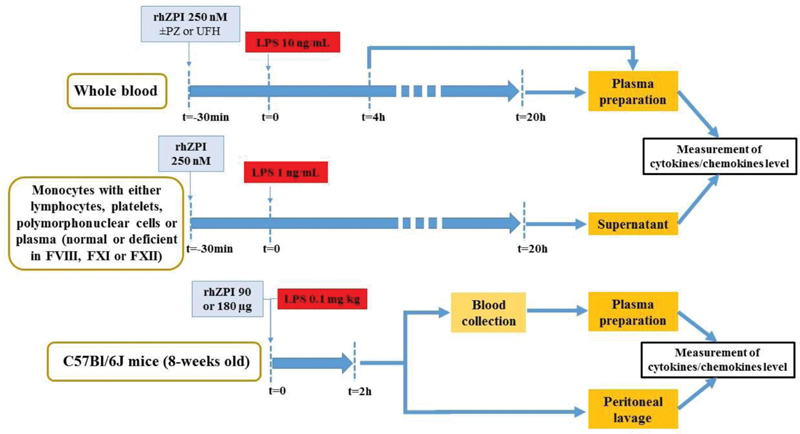
Design of the study. Effects of recombinant human ZPI (rhZPI) was analysed either in whole blood or on isolated fractions of blood (monocytes alone, monocytes and lymphocytes, in the presence or not of platelets or polymorphonuclear cells, and with normal plasma or coagulation factor deficient plasmas). Lipopolysaccharide (LPS) from *E. coli* 0111:B4 was used to stimulate monocytes or to induce *in vivo* inflammation

It is now evident that acute-phase reactants play a role in minimizing the harmful effects of inflammation in host tissue. For example, α-1 antitrypsin (AAT) is an acute-phase protein belonging to the serpin superfamily. 8 The importance in its role is highlighted by the development of emphysema and other inflammatory conditions in individuals with AAT deficiency. 9 It has been suggested that the increase in AAT levels during acute inflammation may not only be protective to the host through blocking excessive serine proteinase activity, but also by regulating the expression of proinflammatory cytokines. 10 Therefore, this prompted us to analyze whether ZPI, in addition to its coagulation regulatory activity, could have a role in dampening excessive inflammatory response, which would be of particular interest in septic shock patients. Effects of recombinant human ZPI (rhZPI) was tested both in vitro and in vivo; Fig. 1 summarizes the different approaches used.

## Materials and Methods

### Reagents

Lipopolysaccharide (LPS) was from *Escherichia coli* 0111:B4 (Sigma-Aldrich, Lyon, France). Purified PZ was from Hyphen-Biomed (Neuville-sur-Oise, France). After reconstitution, it was dialyzed against phosphate buffer saline (PAN Biotech, Germany). UFH was from Sanofi-Aventis (France). Purified FXIa and FXa were from Enzyme Research Laboratories (Stago-BNL, The Netherlands). Congenital deficient plasmas were from George King Bio-medical (Cryopep, France).

### Production and Purification of Recombinant Human ZPI and ZPI (Y387A)

Human ZPI cDNA (NCBI, NM_001100607.2) was cloned into the Not1 and BamH1 site of pCEP4 vector (NeoBiotech, Nanterre, France). ZPI Y387A cDNA was provided by ProteoGenix (France), PCR-base site-directed mutagenesis with pCEP4-ZPI (WT) as a template was used to change the codon for Y387 (TAT) of ZPI into that of alanine (GCT), and mutation was confirmed by sequencing the ZPI cDNA. Then, pCEP4-ZPI (WT) and pCEP4-ZPI (Y387A) were amplified in One Shot Top10 *E. coli* (ThermoFisher, France) according to the manufacturer's instructions.

FreeStyle 293-F cells (Invitrogen, France), grown in F17 medium supplemented with 2 mM glutamine, 0.1 U/mL penicillin, and 0.1 mg/mL streptomycin, were transfected with pCEP4-ZPI (WT) or pCEP4-ZPI (Y387A) by liposome-mediated transfection. Briefly, 200 mL of cells were transfected with 200 µg of plasmid DNA by polyethylenimine on Optimem medium. After 7 days of cells cultured in supplemented F17 medium, the media were collected and centrifuged to remove cells and supernatants were purified.

rhZPI WT and rhZPI (Y387A) were purified similarly from media by two successive chromatography steps on Heparin-Sepharose (GE Healthcare, France) and ResourceQ (GE Healthcare). Briefly, 600 mL of media containing the rhZPI was applied at a flow rate of 5 mL/min to a 5 mL Heparin-Sepharose column equilibrated in 0.1 M NaCl, 0.02 M MES, 1 mM EDTA, pH 6.3, at 20°C. The column was washed with 50 mL equilibrium buffer and then eluted with 20 mL linear gradient from 0.1 to 0.6 M NaCl in the same buffer.

Fractions corresponding to the peak (between 0, 3, and 0.4 M of NaCl) on chromatograph were pooled and dialyzed against 0.015 M NaCl, 0.02 M MES, 0.001 M EDTA, pH 7.4, at 4°C. Dialyzed pooled fractions from previous step were applied at a flow rate of 1 mL/min to a 1 mL ResourceQ column: dialyzed pooled fractions from previous step was applied at a flow rate of 1 mL/min to a 1 mL ResourceQ column equilibrated in 0.02 M NaCl, 0.02 M MES, 1 mM EDTA, pH 7.4, at 20°C. The column was washed with at least 10 mL of the same equilibrium buffer and eluted with 15 mL linear gradient with the same buffer containing 140 mM NaCl.

Fractions were selected by sodium dodecyl sulfate polyacrylamide gel electrophoresis and pooled fractions containing pure rhZPI were concentrated by ultrafiltration with a 30 kDa cutoff membrane on phosphate buffered saline (PBS) buffer. The inhibitory activities of rhZPI against FXIa and FXa in the presence of cofactors were evaluated to validate the batch.

### Whole Blood Assay

Peripheral blood was collected from healthy donors with their informed consent, 9 volumes into 1 volume of 3.2% trisodium citrate (BD vacutainer tubes). Blood collected was diluted (1:1) in RPMI-1640 medium (ThermoFisher Scientific) supplemented with trisodium citrate (3.2%).

A total of 90 µL of diluted blood was incubated for 30 minutes at 37°C and in a 5% CO _2_ humid atmosphere with the different proteins tested, in a volume of 10 µL in a 96-well cell culture plate (VWR, France), before the stimulation by LPS (5 µL, at a concentration of 10 ng/mL). After a 4- or 20-hour incubation period at 37°C, whole blood was collected and centrifuged (2,000 g, 10 minutes) and supernatants were stored at −20°C until cytokine dosages.

### Isolations of Monocytes, Platelets, and Polymorphonuclear Neutrophils

Peripheral blood was depleted in platelets by centrifugation at a low speed (300 g, 10 minutes at room temperature), and platelet-rich-plasma was discarded, then peripheral blood mononuclear cells (PBMCs) were isolated from platelet-depleted-blood by Ficoll-Paque PLUS centrifugation (GE Healthcare) at 300 g for 45 minutes. Cells were washed in RPMI 1640 medium, then resuspended in RPMI-1640 medium supplemented with 10% fetal bovine serum (Gibco, France), 2 mM glutamine, 100 U/mL penicillin, and 100 µg/mL streptomycin (ThermoFisher, France). In a 96-well cell culture plate, 90 µL of PBMC suspension (10 ^5^ cells/well) was preincubated for 30 minutes with 10 µL of the proteins tested and stimulated with 5 µL of LPS at a final concentration of 1 ng/mL.

Monocytes were isolated from lymphocytes by adherence in 48-well cell culture plates (VWR). Briefly, PBMCs (1.2 × 10 ^6^ cells/well) were incubated for 45 minutes at 37°C in a 5% CO _2_ humid atmosphere, then nonadherent cells were eliminated by pipetting and adherent monocytes were washed two times with cold PBS buffer, as previously described. 11 Purified monocytes were cultured in 270 µL of complete RPMI-1640 medium or RPMI-1640 medium supplemented with autologous plasma (10, 25, or 50%). Monocytes were preincubated for 30 minutes with 30 µL of the proteins tested, and then stimulated with 15 µL of LPS (at a final concentration of 1 ng/mL).

Polymorphonuclear neutrophils (PMNs) were isolated from fresh buffy coats prepared from blood of healthy donors. First, leukocytes were separated from erythrocytes by sedimentation on a separating medium containing 5% Dextran T500 (Pharmacia, Sweden) in 0.9% saline. PMNs were separated from PBMCs by centrifugation on Ficoll-Paque PLUS. Contaminating erythrocytes were removed by hypotonic lysis (H _2_ O), and PMNs were resuspended in Hank's balanced salt solution (ThermoFisher Scientific) supplemented with 0.05% FBS and 10 nM HEPES (ThermoFisher Scientific).

For washed platelet preparation, venous blood was collected in 10% ACD/A buffer (75 mM trisodium citrate, 44 mM citric acid, 136 mM glucose, pH 6) and D-phenylalanyl- l -prolyl- l -arginine chloromethyl ketone PPACK (80 µM), and washed platelets were prepared as described previously. 12

### Proteome Profiler Human Cytokine Array kit (R&D Systems, France)

The kit was used according to manufacturer's instructions. Briefly, while nitrocellulose membranes containing different capture antibodies were incubated at room temperature with a blocking buffer, 200 µL of plasma samples was incubated with biotinylated detection antibody to a final volume of 1.5 mL at room temperature for 1 hour. Then, blocking buffer was aspirated and samples were incubated overnight with the membranes at 4°C. Membranes were removed and washed three times for 10 minutes. Streptavidin-HRP was incubated with membranes for 30 minutes and after three washes, captured proteins were visualized using chemiluminescent detection reagents. This cytokine array kit detects simultaneously 28 cytokines and chemokines: IFN-γ, IL-1α, IL-1β, IL-2, IL-4, IL-5, IL-6, IL-8, IL-10, IL-12 p70, IL-13, IL-16, IL-17E, IL-18, IL-21, IL-27, IL-32, MIF, MIP-1α/β, TNF-α, CCL1, CCL2, CCL5, CXCL-1, CXCL-10, CXCL-11, and CXCL12.

### Measurement of Cytokine/Chemokine Levels

Human and murine cytokines (IL-6, TNF-α, MIP-1α, and CCL5) were measured using the specific ELISA Duoset assays (R&D System Bio-techne, Lille, France), according to manufacturer's instructions

### LPS-Induced Inflammation in Mice

Housing and experiments were done as recommended by French regulations and the experimental guidelines of the European Community. This project was approved by the ethical committee CEEA26 under the number APAFIS#5994–201607061607594.v2.

Female WT C57Bl/6J mice (17–20 g; 8 weeks old) were purchased from Envigo (Gannat, France). The animals were maintained in pathogen-free conditions and handled according to national and institutional guidelines.

Inflammation was induced in 8-week-old mice by intraperitoneal injection of LPS (0.1 mg/kg) in NaCl 0.9% (saline) or with rhZPI (90 or 180 µg). Thus, animals were divided into five groups and received either saline ( *n* = 4), rhZPI 90 µg ( *n* = 4), LPS 0.1 mg/kg ( *n* = 9), LPS and 90 µg ZPI combination ( *n* = 8), or LPS and 180 µg ZPI combination ( *n* = 5). Blood was collected before and 2 hours after LPS administration on retro-orbital and intracardiac ways, respectively. Plasma was separated by centrifugation (20 minutes; 1,500 g) and stored at −20°C until analysis. Peritoneal lavage was performed with 2 mL PBS/1% BSA 2 hours after LPS administration.

### Statistical Analysis

Graphpad Prism 6 was used for statistical tests. Repeated measures were analyzed with ordinary ANOVA (analysis of variance) including Dunnett or Bonferroni posttest for multiple comparisons. Student's *t* -test was used when only two groups of mice were compared each other. A *p* -value less than 0.05 was considered statistically significant.

## Results

rhZPI inhibits the synthesis of IL-1β, IL-6, TNF-α, and macrophage inhibitory protein-1 (MIP-1) but increases the production of the chemokine CCL5 (RANTES, Regulated upon Activation, Normal T cell Expressed and Secreted).

**Fig. 2:**
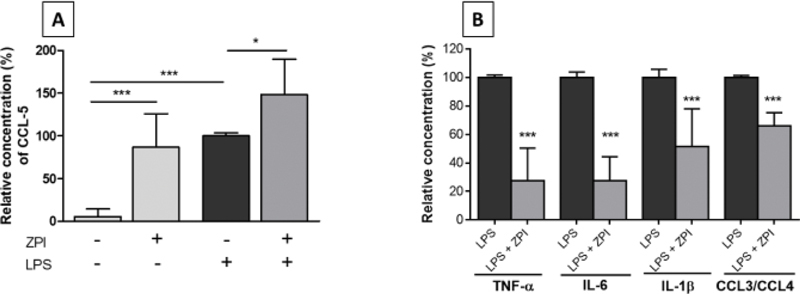
Variations of the production of different inflammatory mediators in whole blood incubated for 4 hours in the presence of PBS (negative controls), recombinant human ZPI (rhZPI, 250 nM), lipopolysaccharide (LPS, 10 ng/mL) or both LPS and ZPI. rhZPI was added 30 min before the addition of LPS. Dots of the microarrays were integrated, according to the manufacturer’s instructions. Results are expressed as percentages of cytokines/chemokines induced by LPS which were considered arbitrarily as 100%. Results (mean ± 1 SEM) are the average of 3 independent experiments. ( **A** ): variations of CCL5 (RANTES), ( **B** ): inhibitory effects of rhZPI on different cytokines/chemokines. The antibody used in the array recognize both CCL3 and CCL4 (MIP-1α and β, Macrophage Inhibitory Protein-1α and β) ^*^ *p* < 0.05, ^***^ *p* < 0.001 as compared to the production of cytokines in the presence of LPS alone [Dunnett’s multiple comparisons test following one-way ANOVA ( **A** ) or Student’s *t* -test ( **B** )].

To identify a possible effect of rhZPI on the production of mediators of inflammation in LPS-stimulated whole blood, we used a protein microarray which allows the simultaneous detection of 36 mediators of inflammation. Whole blood was incubated for 4 hours in the absence of LPS (negative controls), LPS (10 ng/mL) or rhZPI (250 nM) alone, and both LPS and ZPI. In controls, no cytokines or chemokines were detected by the array, except traces of MIF and CCL5, but surprisingly, ZPI alone induced a significant increase of CCL5 ( Fig. 1 ). In the presence of LPS, a significant production of IL-1β, IL-6, TNF-α, IL-1RA, MIF, MIP-1, and CCL5 was observed. The production of IL-1β, IL-6, TNF-α, and MIP-1 was significantly decreased in the presence of rhZPI, whereas the induction of CCL5 by LPS was enhanced by ZPI ( Fig. 2 ). The secretion of IL-1RA and MIF induced by LPS was unaffected by ZPI.

### Recombinant Human ZPI Dose-Dependently Decreases the Production of IL-6 and TNF-α in LPS-Stimulated Whole Blood

**Fig. 3:**
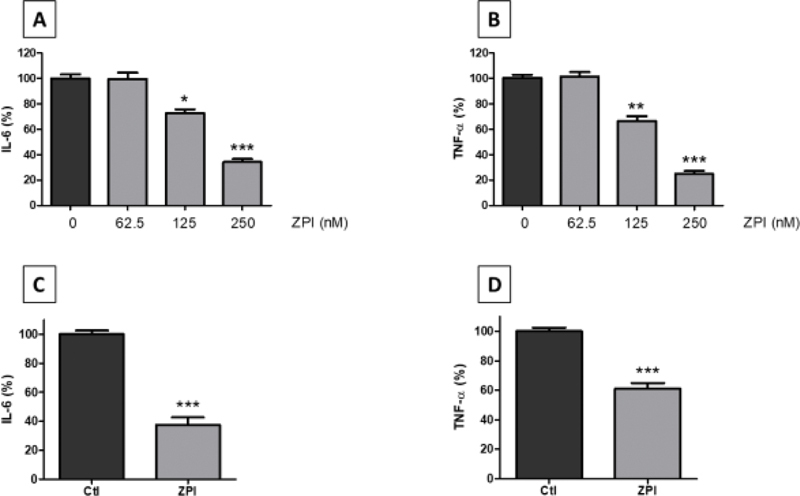
Effects of recombinant human ZPI (rhZPI) on the secretion of IL-6 and TNF-α in whole blood activated by lipopolysaccharide (LPS). Cytokines were quantified after a 20-hour ( **A, B** ) or 4-hour ( **C, D** ) incubation. In the latter case, rhZPI was added at 250 nM. rhZPI was added 30 min before the addition of LPS (10 ng/mL). Results are expressed as percentages of IL-6 or TNF-α production in the absence of rhZPI (controls) which were considered arbitrarily as 100%. Results (mean ± 1 SEM) are the average of 3 ( **A, B** ) or 4 ( **C, D** ) independent experiments in triplicate. ^*^ *p* < 0.05, ^**^ *p* < 0.01, ^***^ *p* < 0.001 as compared to the production of cytokines in the presence of LPS alone [Dunnett’s multiple comparisons test following one-way ANOVA ( **A, B** ) or Student’s *t* -test ( **C, D** )].

To confirm and characterize the inhibitions of the proinflammatory cytokines IL-6 and TNF-α observed in the microarray, increasing concentrations of rhZPI (from 62.5 to 250 nM, corresponding to 1- to 4-fold the physiological concentrations of plasma ZPI) were added in whole blood, 30 minutes before the addition of LPS (10 ng/mL). After a 20-hour incubation, plasma levels of IL-6 and TNF-α were quantified. IL-6 and TNF-α were dose-dependently decreased, with a significant inhibitory effect ( *p* < 0.05) observed from 125 nM of rhZPI ( Fig. 3A , B ). This inhibitory effect was also observed when a shorter incubation time (4 hours) was chosen ( Fig. 3C , D ).

### Effects of Purified PZ or UFH on the Inhibitory Effect of rhZPI on the Production of IL-6 and TNF-α in LPS-Stimulated Whole Blood

As ZPI forms a complex with PZ, and as some biological effects of ZPI are potentiated by PZ (for example, the inhibition of FXa), we evaluated if the downregulation of IL-6 and TNF-α in LPS-stimulated whole blood was dependent on the presence of PZ. Increasing concentrations of purified PZ alone, from 62.5 to 250 nM, had no significant effect on the production of IL-6 or TNF-α ( Table 1 ). At the lowest concentration tested (62.5 nM), rhZPI alone has no significant inhibitory effect on the production of IL-6 or TNF-α, but in the presence of an equimolecular concentration of PZ (62.5 nM), a significant inhibitory effect was observed on the secretion of TNF-α ( Table 1 ). For the higher concentrations of rhZPI, the addition of PZ did not further increase the inhibitory effect of ZPI.

**Table 1:** Effects of purified protein Z (PZ) on the inhibitory effects of recombinant human ZPI (rhZPI) on the secretion of IL-6 and TNF-α in LPS-stimulated whole blood

Conc. (nM)	IL-6 inhibition (%)				TNF-α inhibition (%)			
	PZ	ZPI	PZ and ZPI	*p* (ZPI vs. PZ and ZPI)	PZ	ZPI	PZ and ZPI	*p* (ZPI vs. PZ and ZPI)
62.5	5.7 ± 0.9	0.5 ± 0.9	19.9 ± 3.9 a	0.001	0.23 ± 9	1.2 ± 5	30.5 ± 2.3 a	0.006
125	17.4 ± 14.1	27.6 ± 3.9 b	35.1 ± 2.2	0.24	11.6 ± 4.5	33.7 ± 4.5 c	50.2 ± 9.7	0.185
250	21.8 ± 6	65.8 ± 3 d	71.6 ± 1.9	0.437	3.2 ± 7.8	75.1 ± 3.2 d	82.2 ± 6.8	0.786

Abbreviations: IL-6, interleukin-6; LPS, lipopolysaccharide; TNF-α, tumor necrosis factor-α; ZPI, Z-dependent plasma protease inhibitor.

Note: rhZPI and PZ, at equimolecular concentrations (Conc.), were added 30 minutes before the addition of LPS (10 ng/mL). Results are expressed as percentages of inhibition of IL-6 or TNF-α production. Results (mean ± 1 SEM) are the average of three independent experiments in triplicate.

a *p* < 0.01 as compared with the effect of ZPI alone.

b *p* < 0.05 (as compared with the production of cytokines in the absence of ZPI).

c *p* < 0.01 (as compared with the production of cytokines in the absence of ZPI).

d *p* < 0.001 (as compared with the production of cytokines in the absence of ZPI).

It has been observed that UFH increases the inhibitory capacity of ZPI toward FXIa. Therefore, we analyzed whether the addition of UFH influences the inhibitory effects of rhZPI on the production of IL-6 and TNF-α in LPS-stimulated whole blood. Increasing concentrations of UFH up to 0.6 U/mL had no significant effect on the inhibitory effect of rhZPI (data not shown).

### The Inhibitory Effect of ZPI on the Production of IL-6 and TNF-α in LPS-Stimulated Whole Blood Is Conserved Using rhZPI (Y387A)

**Fig. 4:**
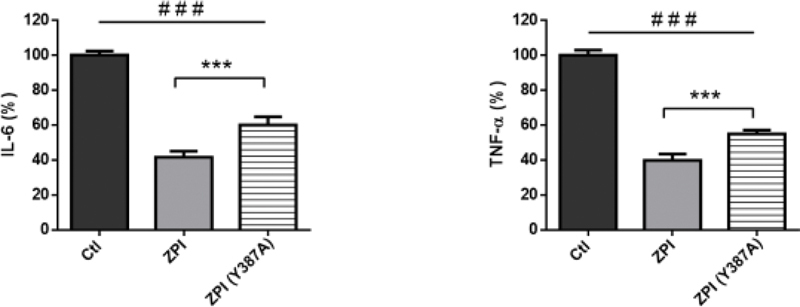
Effects of the rhZPI-Y387A variant on the secretion of IL-6 and TNF-α in whole blood activated by lipopolysaccharide (LPS). Comparison with wild-type rhZPI (ZPI) effects. Cytokines were quantified after a 20 hours incubation. rhZPI-Y387A or rhZPI were added at 250 nM 30 min before the addition of LPS (10 ng/mL). Results are expressed as percentages of IL-6 or TNF-α production in the absence of ZPI (controls) which were considered arbitrarily as 100%. Results (mean ± 1 SEM) are the average of 6 independent experiments in triplicate. ^###^ *p* < 0.001 as compared to the production of cytokines in the presence of LPS alone, ^***^ < 0.001 as compared to the production of cytokines in the presence of wild-type ZPI (Dunnett’s multiple comparisons test following one-way ANOVA).

Tyrosine 387 has been shown to be the P(1) residue at the reactive center of the ZPI molecule and rhZPI(Y387A), a form of ZPI in which tyrosine 387 has been changed to alanine, lacks PZ-dependent FXa and FXIa inhibitory activity. 1 2 To analyze the importance of the reactive center loop (RCL) of ZPI, we studied the effects of the rhZPI(Y387A) on the proinflammatory cytokine production in LPS-stimulated whole blood. As shown on Fig. 4 , rhZPI(Y387A) had a significant inhibitory effect on the production of IL-6 and TNF-α induced by LPS. The inhibitory effect of the variant was slightly weaker than the inhibitory effect of rhZPI. The rhZPI(Y387A) also inhibited the secretion of proinflammatory cytokines when the incubation period was decreased to 4 hours (data not shown). Taken together, these data suggest that the RCL contributes in part to the anti-inflammatory activity of rhZPI, but that most of anti-inflammatory activity is independent of the RCL.

### The Inhibitory Effects of rhZPI Is Observed Even When It Is Added after LPS Stimulation

**Fig. 5:**
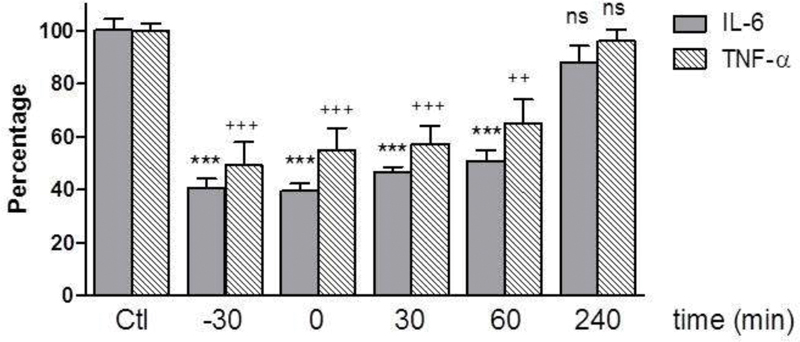
Inhibitory effects of 250 nM of recombinant human ZPI (rhZPI) according to the delay within lipopolysaccharide (LPS) is added to the whole blood. rhZPI was added 30 min before, simultaneously or 30, 60 and 240 min after the addition of LPS (10 ng/mL). Results are expressed as percentages of IL-6 or TNF-α production in the absence of rhZPI (controls) which were considered arbitrarily as 100%. Results (mean ± 1 SEM) are the average of 3 independent experiments in triplicate. ^***^ *p* < 0.001 as compared to the production of IL-6 in the presence of LPS alone. ^++^ < 0.01, ^+++^ < 0.001 as compared to the production of TNF-α in the presence of LPS alone (Dunnett’s multiple comparisons test following one-way ANOVA).

In our first experiments, rhZPI was added before the stimulation by LPS. Therefore, we checked whether ZPI could have an inhibitory effect even after the stimulation of blood by LPS. As it can be seen in Fig. 5 , the inhibitory effect of ZPI on the production of IL-6 and TNF-α on LPS-stimulated whole blood was still significant when ZPI was added 1 hour after the addition of LPS. This effect disappeared when ZPI was added 4 hours after LPS stimulation.

### The Inhibitory Effect of ZPI on Cytokine Production by LPS-Activated Monocytes Requires a Cellular Cooperation and the Presence of Plasma

**Fig. 6:**
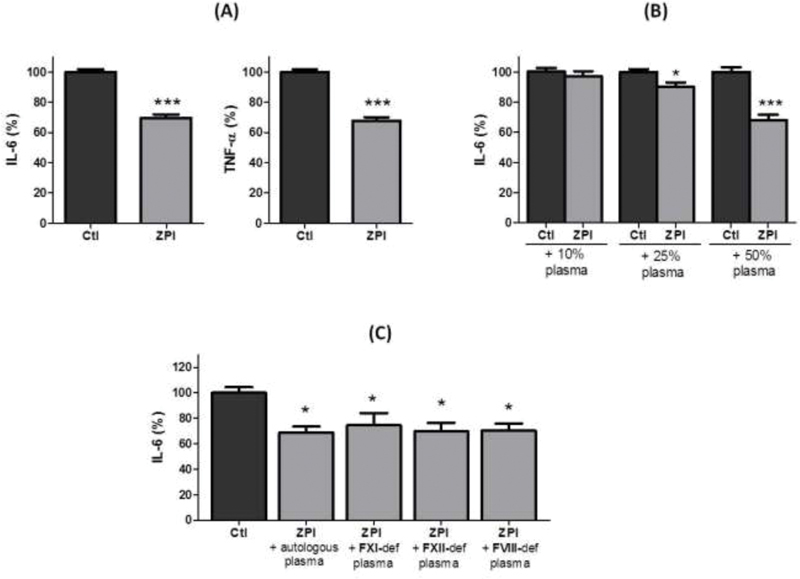
Effects of cellular cooperation and plasma on the inhibitory effects of recombinant human ZPI (250 nM) on isolated lipopolysaccharide (LPS)-stimulated monocytes. rhZPI was added 30 min before the addition of 1 ng/mL of LPS. ( **A** ) Effects of ZPI on peripheral blood mononuclear cells. ( **B** ) Variations of IL-6 production by LPS-activated monocytes in the presence of ZPI (250 nM) and increasing concentrations of normal plasma. ( **C** ) Variations of IL-6 production by LPS activated monocytes in the presence of ZPI (250 nM) and increasing concentrations of plasmas deficient in coagulation factors. Results are expressed as percentages of cytokines induced by LPS which were considered arbitrarily as 100%. Results (mean ± 1 SEM) are the average of 3 independent experiments in triplicate. ^*^ *p* < 0.05, ^***^ *p* < 0.001 as compared to the production of cytokines in the presence of LPS alone [Student’s *t* -test ( **A, B** ) or Dunnett’s multiple comparisons test following one-way ANOVA ( **C** )].

To better characterize the mechanisms of the inhibitory effect of rhZPI on the production of proinflammatory cytokines induced by LPS, we analyzed the effects of rhZPI on purified cells. Surprisingly, ZPI was unable to inhibit the production of IL-6 or TNF-α by purified monocytes stimulated with a weak concentration (1 ng/mL) of LPS ( Supplementary Fig. 1 ). The inhibitory effect of rhZPI was only present on PBMCs ( Fig. 6A ), suggesting that a cooperation between lymphocytes and monocytes is needed. In contrast, rhZPI was inefficient to inhibit the cytokine production induced by LPS when monocytes were co-incubated with washed platelets or purified polymorphonuclear cells (data not shown). Finally, we investigated whether the inhibitory effect of rhZPI was dependent on the presence of plasma: a weak, but significant, inhibitory effect was observed on IL-6 production by LPS-stimulated monocytes ( Fig. 6B ), whereas TNF-α production was unaffected. As ZPI alone, in the absence of PZ, inhibits only FXIa, we tested if the decrease of IL-6 production was related to the inhibition of FXIa. However, the inhibitory effect of rhZPI was still observed when FXI-deficient plasma was used instead of normal plasma. An inhibitory effect of rhZPI on IL-6 production was also observed when XII- or FVIII-deficient plasmas were used ( Fig. 6C ).

### Administration of Recombinant Human ZPI to Mice Inhibits the Increase of Plasma Levels of IL-6 and TNF-α Induced by Intraperitoneal Injection of LPS

**Fig. 7:**
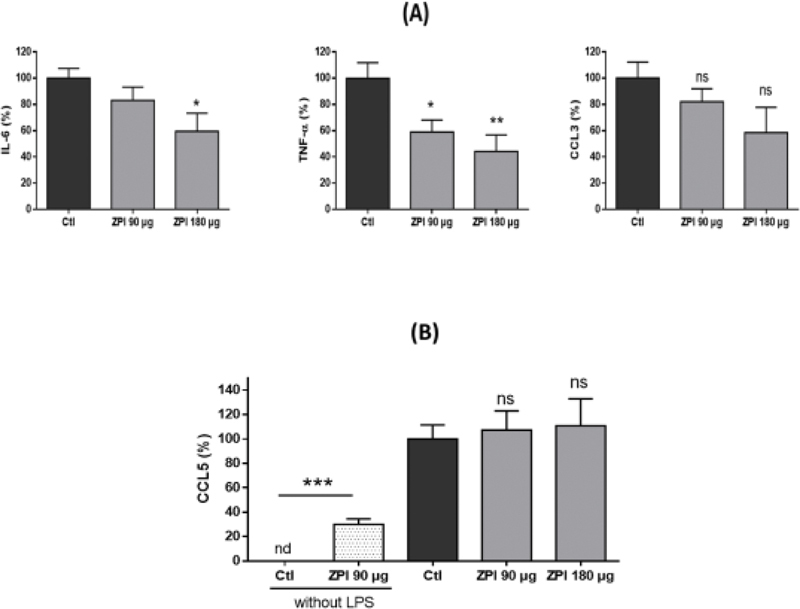
Effects of rhZPI administration (90 or 180 μg) on lipopolysaccharide (LPS)-induced IL-6, TNF-α, CCL3 and CCL5 production in mice. ( **A** ): variations of IL-6, TNF-α and CCL3 levels, ( **B** ) variations of CCL5 in peritoneal lavage. Mice received intraperitoneal injection of either saline (negative control, *n* = 9), ZPI 90 μg ( = 4), lipopolysaccharide (LPS) 0.1 mg/kg alone ( = 9), LPS and 90 μg ZPI ( = 8), or LPS and 180 μg ZPI ( = 5). Plasma and peritoneal lavages were collected 2 hours after LPS administration. ( **A** ): variations of IL-6, TNF-α and CCL3 levels. Controls (injection of LPS alone) were considered arbitrarily as 100%. ( **B** ) variations of CCL5 in peritoneal lavages. ^*^ *p* < 0.05, ^**^ *p* < 0.01, ^***^ *p* < 0.001 (Dunnett’s multiple comparisons test following one-way ANOVA). nd, not detected; ns, not significant.

We also evaluated whether the in vivo administration of ZPI to mice could counterbalance the inflammatory response induced by LPS. Mice were injected intraperitoneally (i.p.) with 0.1 mg/kg of LPS, alone or in combination with 90 or 180 µg of rhZPI. A significant decrease of plasma TNF-α was observed at the dose of 90 µg; plasma IL-6 was only decreased at the highest dose tested ( Fig. 7A ). There was a trend toward a decrease of plasma CCL3, which was not statistically significant. As observed in the whole blood model, i.p. injection of ZPI induced a significant secretion of CCL5 in peritoneal lavage, but it did not enhance the production of CCL5 induced by LPS alone ( Fig. 7B ).

## Discussion

In the present work, we describe that the serpin ZPI exerts an anti-inflammatory activity, both in vitro and in vivo. This effect requires high concentrations of ZPI, since, in vitro, the maximal effect on IL-6 and TNF-α was observed at a fourfold physiological concentration. The serpin antithrombin (AT) has been shown to exert anti-inflammatory activity in vitro at very high concentrations (20 UI/mL), 13 but in vivo *,* the high doses required to reverse organ dysfunction in sepsis are associated with an increased bleeding tendency. 14 Similarly, in vitro, activated protein C (aPC) was shown to have anti-inflammatory properties at 20-fold the physiological concentration of protein C, 15 but its use in clinic at high doses has been associated with an excessive hemorrhagic risk. 16 Therefore, as the cytoprotective and anti-inflammatory effects of the physiological coagulation inhibitors (AT and aPC) appear at much higher concentrations than their anticoagulant properties, their use in clinical situations is limited. The major interest of ZPI is that, in the absence of PZ, it inhibits only FXIa, the inhibition of which does not cause spontaneous bleeding disorder. 17 Interestingly, the inhibition of FXIa generation was shown to improve the survival of mouse in a model of polymicrobial sepsis, and to minimize the inflammatory response as well as the coagulopathy. 18 Taken together, these preliminary data suggest that ZPI could constitute an adjuvant therapy in the treatment of sepsis, since, in contrast to the other natural anticoagulants hitherto tested, the high doses required to exhibit an anti-inflammatory activity are unlikely associated with a bleeding tendency.

Several reports suggested a link between the activation of the contact phase of the coagulation and inflammation. Activated FXII (FXIIa) was recently shown to induce the production of proinflammatory cytokines by macrophages, 19 and it was precedingly indicated that the inhibition of FXIa generation by the antibody 14E11 decreased circulating levels of IL-6 and TNF-α. 18 Therefore, it could be speculated that the anti-inflammatory activity of high concentrations of rhZPI could be due to the inhibition of FXIa. However, this seems unlikely, since the anti-inflammatory activity of rhZPI was conserved even in FXI-deficient plasma and since the variant of ZPI (Y387A) which has lost its inhibitory activity toward FXIa still has an anti-inflammatory activity. It is to be noted that the variant (Y387A) was slightly less efficient than the WT rhZPI to inhibit the production of proinflammatory cytokines by LPS-activated monocytes. Therefore, we cannot totally exclude a participation of the reactive loop center of rhZPI to explain its anti-inflammatory capacity, but most of the anti-inflammatory activity of rhZPI is independent of the active center.

The mechanism of anti-inflammatory activity of ZPI is unclear. For AT and aPC, for example, it was shown that they inhibit the nuclear translocation of nuclear factor kappa B (NF-κB) induced by LPS, and which is necessary to induce the secretion of IL-6 and TNF-α by monocytes. 13 15 The anti-inflammatory activity of these proteins was detected on purified monocytes. In contrast, we were unable to show an inhibitory activity of ZPI on purified monocytes stimulated by LPS as well as an impairment of NF-κB translocation to the nucleus (data not shown). An inhibitory effect of ZPI was evidenced on PMBCs stimulated by LPS, suggesting that a cooperation between both cellular types is mandatory for rhZPI to exert an anti-inflammatory activity. Surprisingly, we observed that incubation of ZPI alone induced a rapid secretion of CCL5 (RANTES). CCL5 is stored in high quantity in platelets and can be released by platelet stimulation. 20 Therefore, we first eliminated the hypothesis, using washed platelets and flow cytometry analysis of platelet activation, that the increase of RANTES was due to a platelet activation by the rhZPI (data not show). CCL5 is mainly produced by lymphocytes 21 and we can suspect that this secretion of CCL5 is necessary to decrease the production of proinflammatory cytokines by LPS-activated monocytes. Indeed, it was recently published that CCL5 efficiently inhibits the release of IL-1β in response to stimulation of ATP receptor present on monocyte surface. 22 In our whole blood model, we observed that recombinant CCL5 decreased significantly the production of IL-6 and TNF-α ( Supplementary Fig. 2 ), giving consistence to a role of the increase of CCL5 induced by ZPI and the concomitant decrease of IL-6 and TNF-α.

An interrelation between the PZ/ZPI complex and the inflammation pathways was previously suggested by the report of Butschkau et al, 23 indicating that levels of IL-6 and IL-10 were substantially and significantly increased in PZ-deficient mice compared with their WT littermates, when they were submitted to a generalized Shwartzman reaction. They did not observe any consequences of ZPI deficiency on cytokine levels. These differences could be due to the fact that in mice, in contrast to humans, there is no free ZPI, since PZ is in excess in comparison to ZPI and all the ZPI is complexed to PZ. Furthermore, human and murine ZPI are quite different, since murine ZPI has a higher molecular mass than human ZPI (92 vs. 72 kDa), suggesting different glycosylations, which could modify the activity of murine protein. In contrast, we were unable to show any capacity of PZ to modulate inflammation.

In conclusion, we have evidenced the capacity of human ZPI to downregulate the inflammation both in vitro and in vivo. As previously observed with AAT, another serpin positively regulated by inflammation, the mechanism(s) involved in its anti-inflammatory activity is not restricted to its active site but clearly deserve further investigations.

## Notes

**What Is Known on This Topic?**

- The protein Z-dependent plasma protease inhibitor (ZPI) is a serpin that inhibits factor XIa and FXa, in the presence of protein Z.
- The physiological importance of ZPI as a physiological anticoagulant is still debated.
- ZPI is an acute-phase protein.

**What Does This Paper Add?**

- ZPI inhibits synthesis of proinflammatory cytokines, both in vitro and in vivo.
- The anti-inflammatory activity of ZPI does not require its active center loop.
