# Supplementary material for: Anti-inflammatory Activity of the Protein Z-Dependent Protease Inhibitor
Source: TH Open. 2021 Jun 25;5(2):e220–9. doi: 10.1055/s-0041-1730037 (PMC8233056; doi:10.1055/s-0041-1730037)
Supplement: Supplementary file 1 — Supplementary Material [file 10-1055-s-0041-1730037-s210020.pdf]

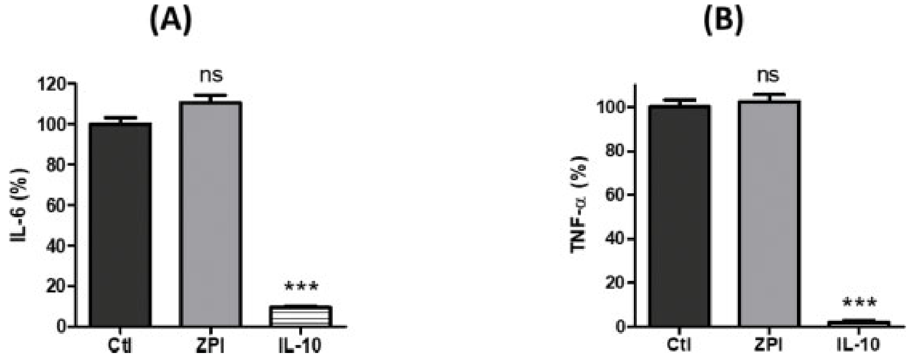

Supplementary Fig. 1 Absence of inhibitory effects of recombinant human ZPI (250 nM) on lipopolysaccahride (LPS)-induced IL-6 (**A**) and TNF-α (**B**) production on isolated monocytes. Recombinant IL-10 was used as positive control. rhZPI or IL-10 (10 ng/mL) was added 30 min before the addition of 1 ng/mL of LPS. Results are expressed as percentages of cytokines induced by LPS which were considered arbitrarily as 100%. Results (mean ± 1 SEM) are the average of 4 independent experiments in triplicate. ns: not significant, $^{***}p < 0.001$ as compared to the production of cytokines in the presence of LPS alone. ns : not significant.

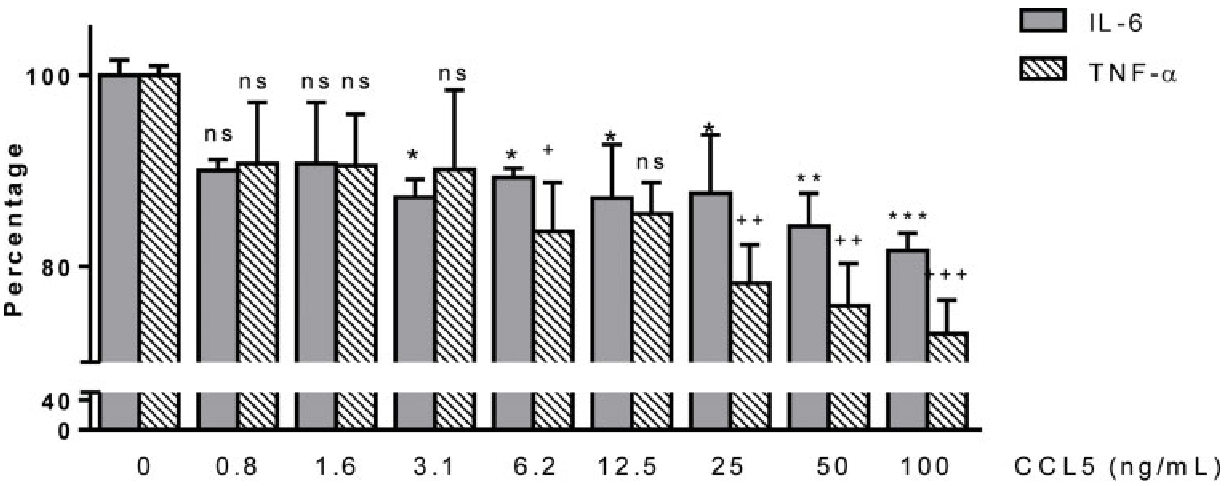

Supplementary Fig. 2 Effects of recombinant human CCL5 on lipopolysaccharide (LPS)-induced IL-6 and TNF-α production in whole blood model. Recombinant human CCL5 was added 30 min before the addition of LPS (10 ng/mL). Results are expressed as percentages of IL-6 or TNF-α production in the absence of CCL5 (controls) which were considered arbitrarily as 100%. Results (mean ± 1 SEM) are the average of 4 independent experiments in triplicate. ns: non significant, $^{*}p < 0.05$, $^{**}p < 0.01$, $^{***}p < 0.001$ as compared to the production of IL-6 in the presence of LPS alone. ns: not significant, $^{+}p < 0\ 0.05$, $^{++}p < 0\ 0.01$, $^{+++}p < 0.001$ as compared to the production of TNF-α in the presence of LPS alone.

 © 2021. The Author(s).
